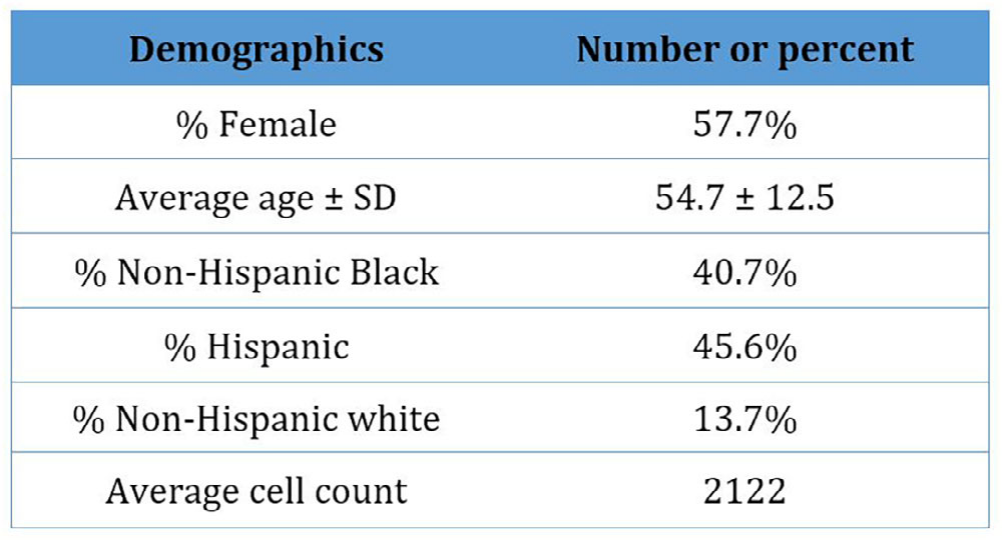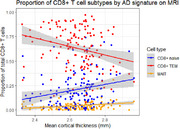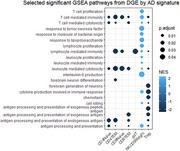# Multi‐omic Immune Cell Phenotypes Associate with Cortical Thickness and Cognition in a Multi‐Ethnic Cohort

**DOI:** 10.1002/alz70855_107352

**Published:** 2025-12-25

**Authors:** Dallin Dressman, Edric D. Winford, Badri N. Vardarajan, Maedot Yidenk, Ryan Talcoff, Jessica Mazen, Elizabeth M Bradshaw, Jennifer J. Manly, Adam Brickman, Wassim Elyaman

**Affiliations:** ^1^ Taub Institute for Research on Alzheimer's Disease and the Aging Brain, Columbia University, New York, NY, USA; ^2^ Department of Neurology, Columbia University, New York, NY, USA; ^3^ Department of Neurology, Vagelos College of Physicians and Surgeons, Columbia University, New York, NY, USA; ^4^ The Gertrude H. Sergievsky Center, College of Physicians and Surgeons, Columbia University, New York, NY, USA; ^5^ The Gertrude H. Sergievsky Center, Vagelos College of Physicians and Surgeons, Columbia University, New York, NY, USA; ^6^ Institute for Genomic Medicine, Columbia University, New York, NY, USA; ^7^ G.H. Sergievsky Center, Vagelos College of Physicians and Surgeons, Columbia University, New York, NY, USA

## Abstract

**Background:**

The immune response to aging and Alzheimer's disease (AD) can be protective against disease in some individuals, and dysregulated and damaging in others. Factors like genetics, socioeconomic status, race, and ethnicity can affect individual risk of AD. Profiling immune phenotypes correlated with aspects of biological and cognitive aging may identify sources of variation in AD risk, and highlight immune processes that can be pharmacologically targeted in individuals at higher risk of AD.

**Methods:**

We performed single‐cell RNA, T, and B cell receptor sequencing of >439,000 circulating immune cells from 205 participants ages 29‐81 (mean 54.7±12.5) in the Offspring Study of Racial and Ethnic Disparities in Alzheimer's Disease, with plasma proteomics data from 86 of the sequencing participants. We correlated immune cell type proportions, T cell clonal expansion, and cell type‐specific gene expression with cognitive scores and mean cortical thickness in brain areas associated with AD, controlling for participant age, sex, and self‐reported race and ethnicity.

**Results:**

Cortical thickness correlated with several immune cell phenotypes. Within the CD8+ T cell pool, proportions of naïve and mucosal‐associated invariant T (MAIT) cells were higher with higher cortical thickness (naïve: r = 0.23, *p* = 0.0065, MAIT: r = 0.24, *p* = 0.0042), while proportions of effector memory cells were lower (*r* = ‐0.26, *p* = 0.0027). Higher cortical thickness was also associated with lower T cell clonal expansion, and with lower expression in several T cell subtypes of genes in biological pathways such as antigen presentation, cytotoxicity, and T cell immunity. Higher total cognitive score also correlated with lower expression of genes related to cytotoxicity, antigen presentation, and antimicrobial defense in gamma‐delta T cells.

**Conclusions:**

In a multi‐ethnic cohort of middle‐aged adults, we showed that higher naïve CD8+ T cell proportions and reduced expression of cytotoxicity‐related genes across T cell subtypes are associated with higher cortical thickness in AD‐relevant brain regions, even after adjusting for age. This finding suggests that therapeutic approaches to maintain the naïve T cell pool and target T cell cytotoxicity and clonal expansion may help protect against atrophy in AD‐related brain regions and boost cognition in older adults.